# Supplementary material for: Following the niche: the differential impact of the last glacial maximum on four European ungulates
Source: Commun Biol. 2022 Sep 29;5:1038. doi: 10.1038/s42003-022-03993-7 (PMC9523052; doi:10.1038/s42003-022-03993-7)
Supplement: Supplementary file 2 — Supplementary Information [file 42003_2022_3993_MOESM2_ESM.pdf]

Supplementary Information for:

Following the niche: the differential impact of the Last Glacial Maximum on four European ungulates

Michela Leonardi[1]*, Francesco Boschin[2]*, Paolo Boscato[2], Andrea Manica[1].

1 Evolutionary Ecology Group, Department of Zoology, University of Cambridge, Downing Street, Cambridge CB2 3EJ, UK.
2 U.R. Preistoria e Antropologia, Dipartimento di Scienze Fisiche della Terra e dell'Ambiente, Università degli Studi di Siena, Via Laterina 8, 53100 Siena, Italy.

*Corresponding authors:
Michela Leonardi (ORCID: 0000-0001-8933-9374; ml897@cam.ac.uk),
Francesco Boschin (ORCID: 0000-0001-5795-9050; francesco.boschin@unisi.it).

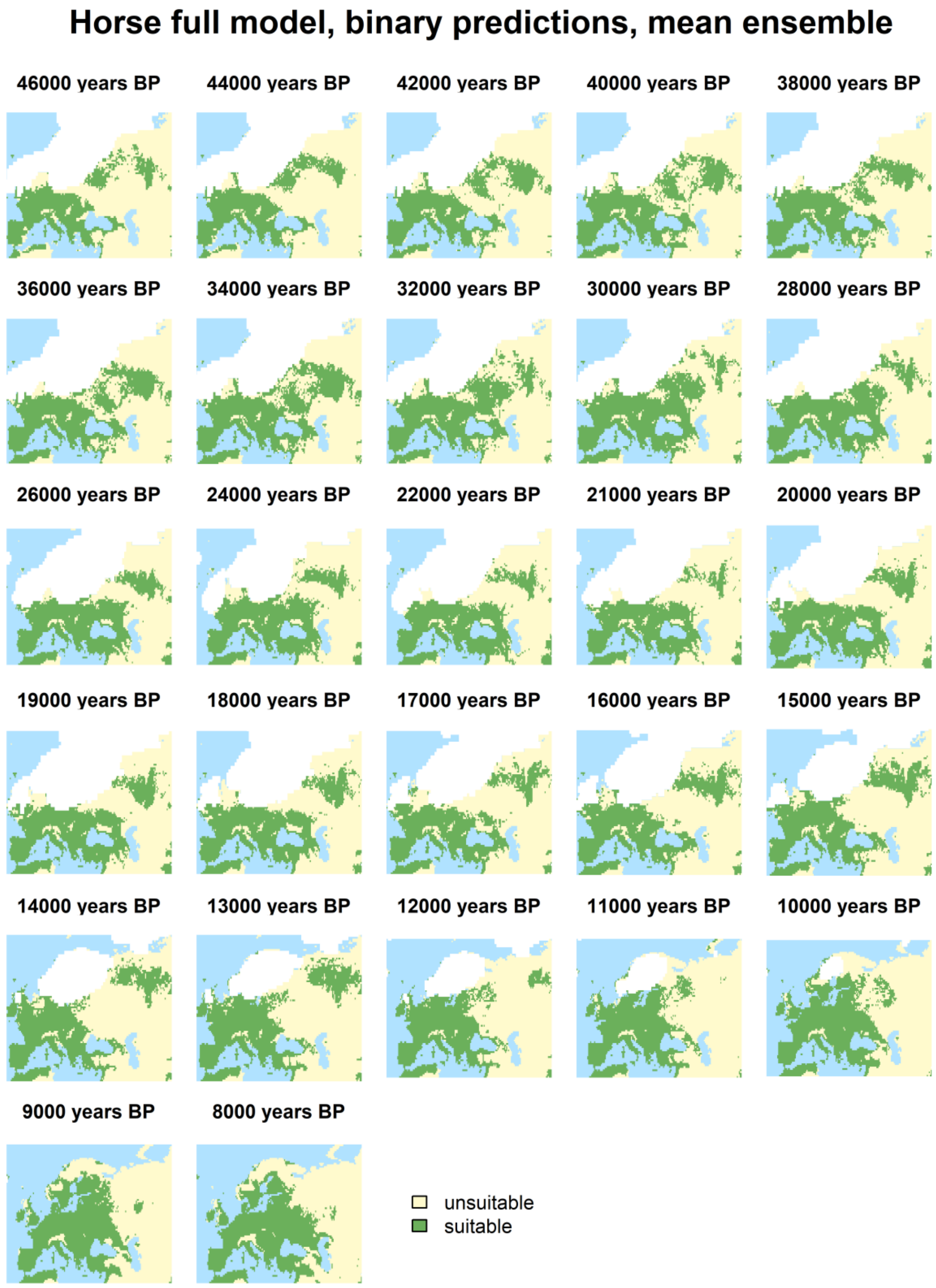

Supplementary figure 1: Projection of the potential distribution of the horse over time based on the median of the changing niche (=full) model ensemble.

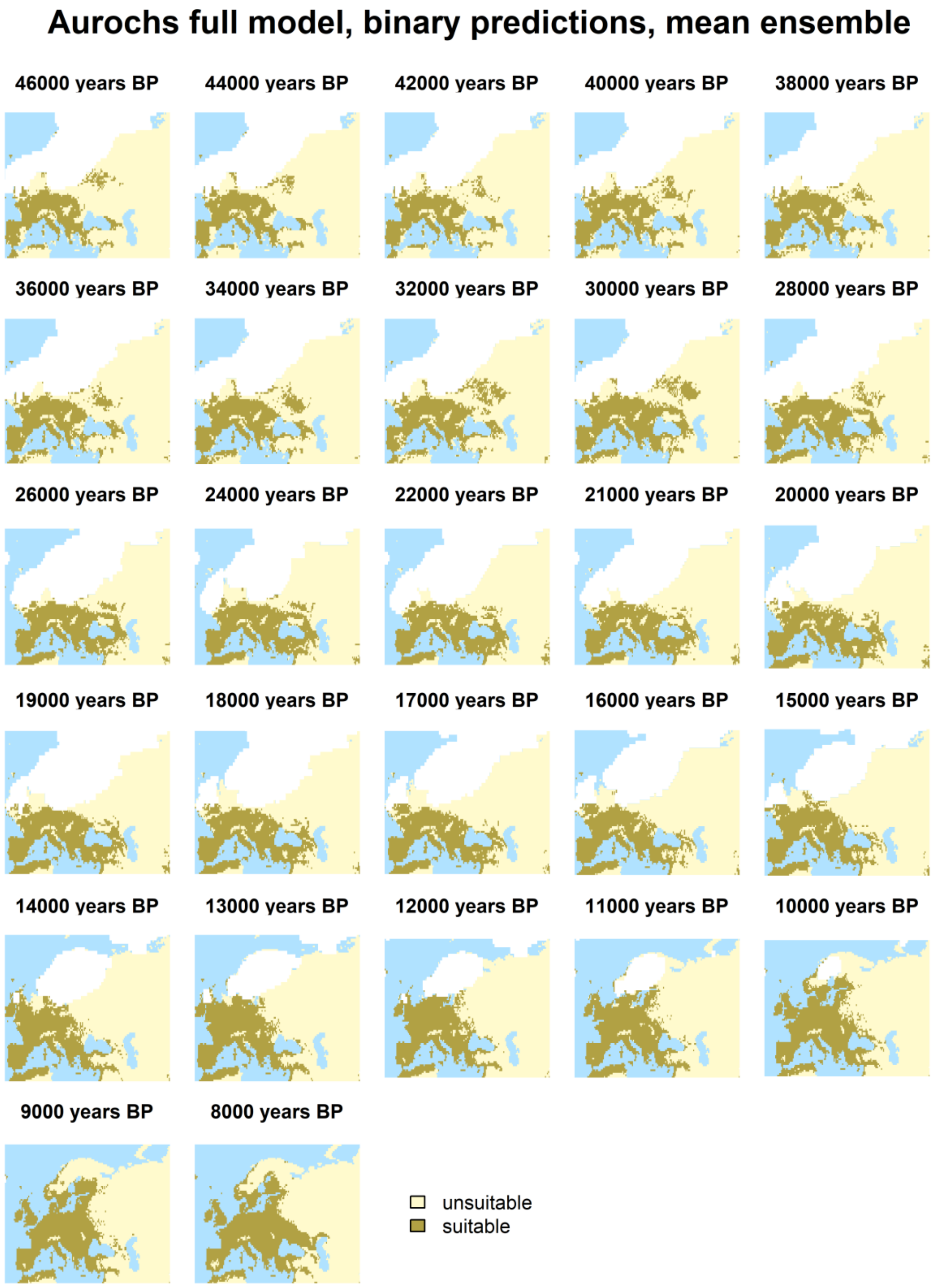

Supplementary figure 2: Projection of the potential distribution of the aurochs over time based on the median of the changing niche (=full) model ensemble.

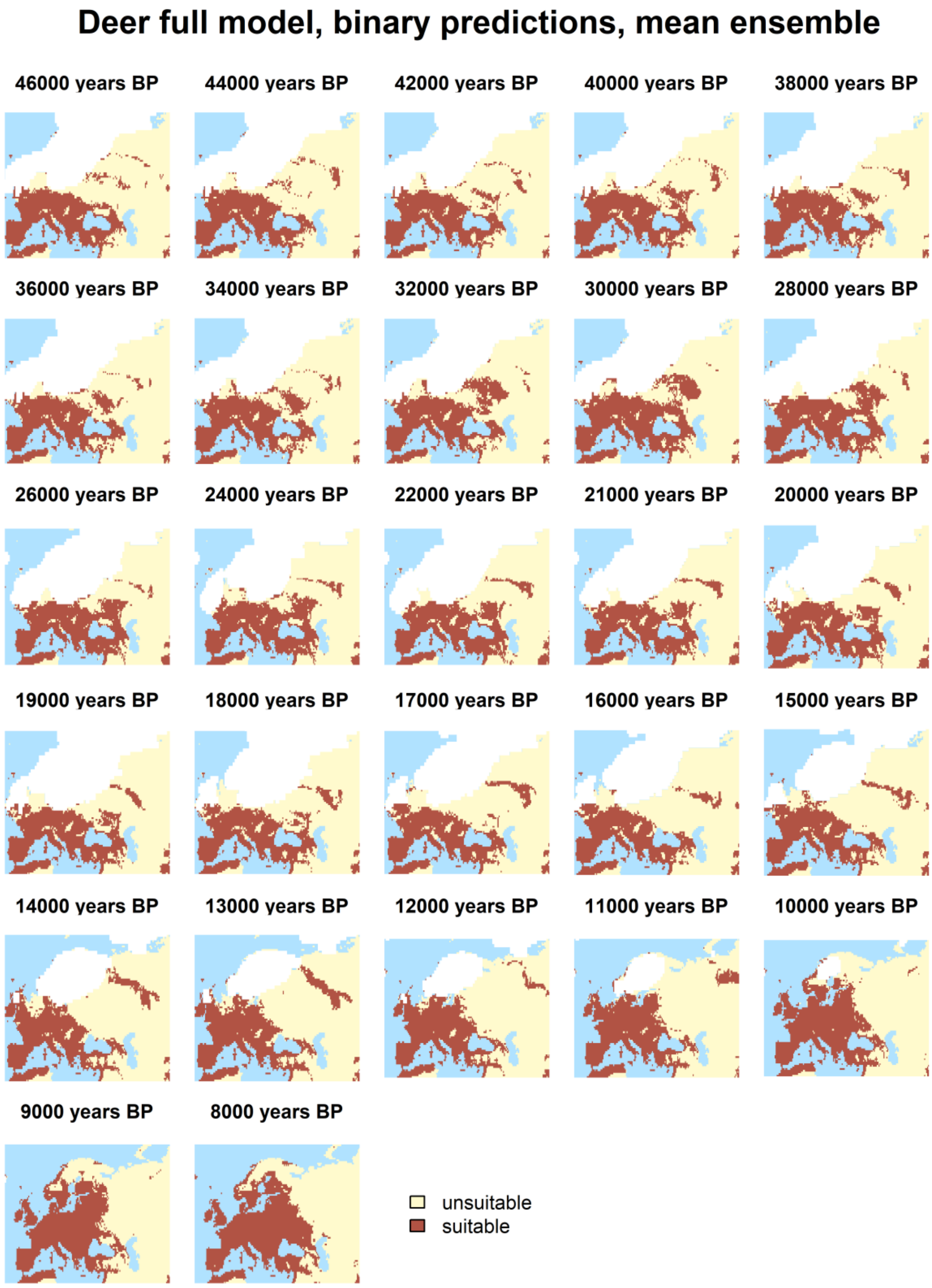

Supplementary figure 3: Projection of the potential distribution of the deer over time based on the median of the changing niche (=full) model ensemble.

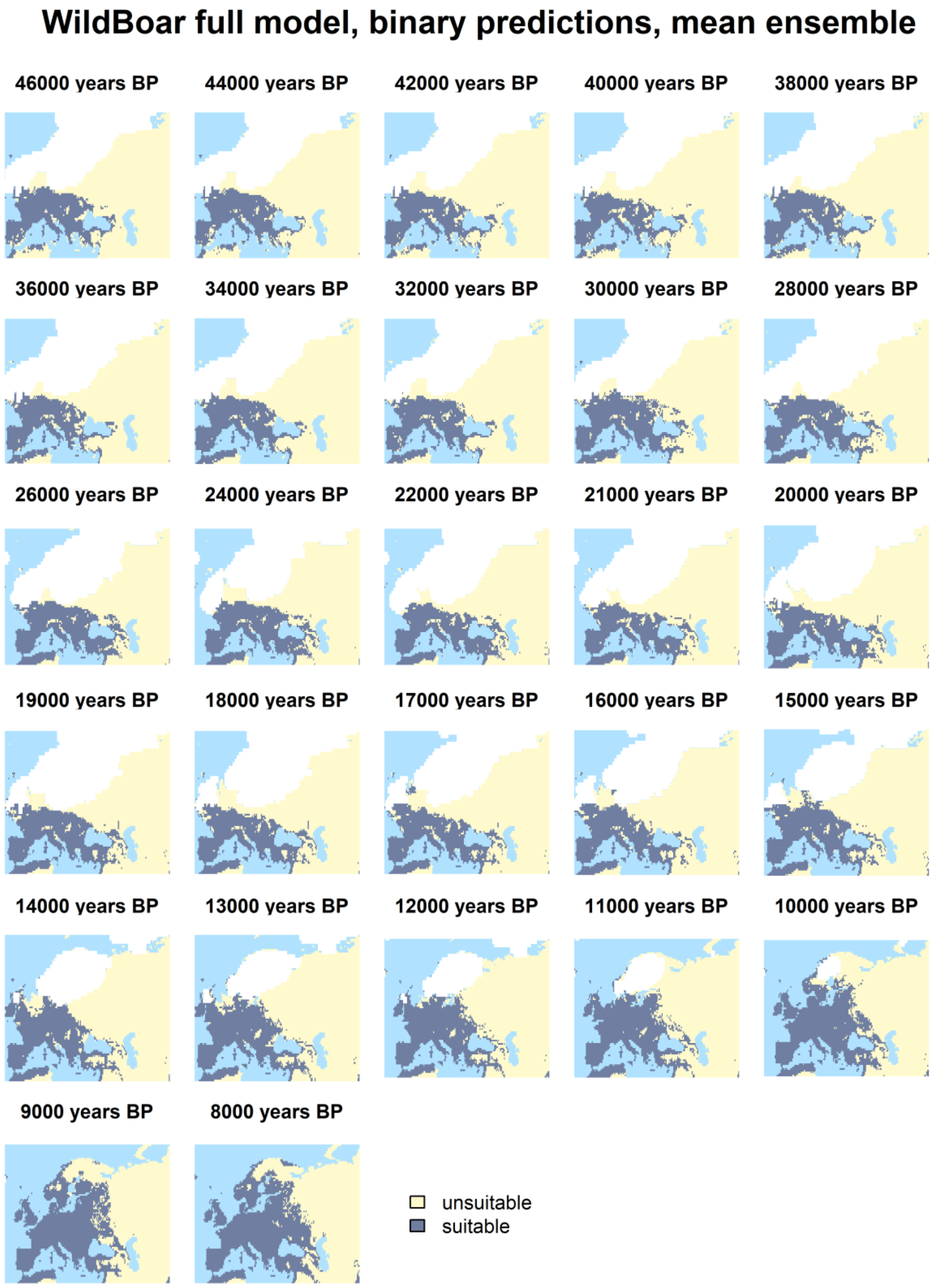

Supplementary figure 4: Projection of the potential distribution of the wild boar over time based on the median of the changing niche (=full) model ensemble.

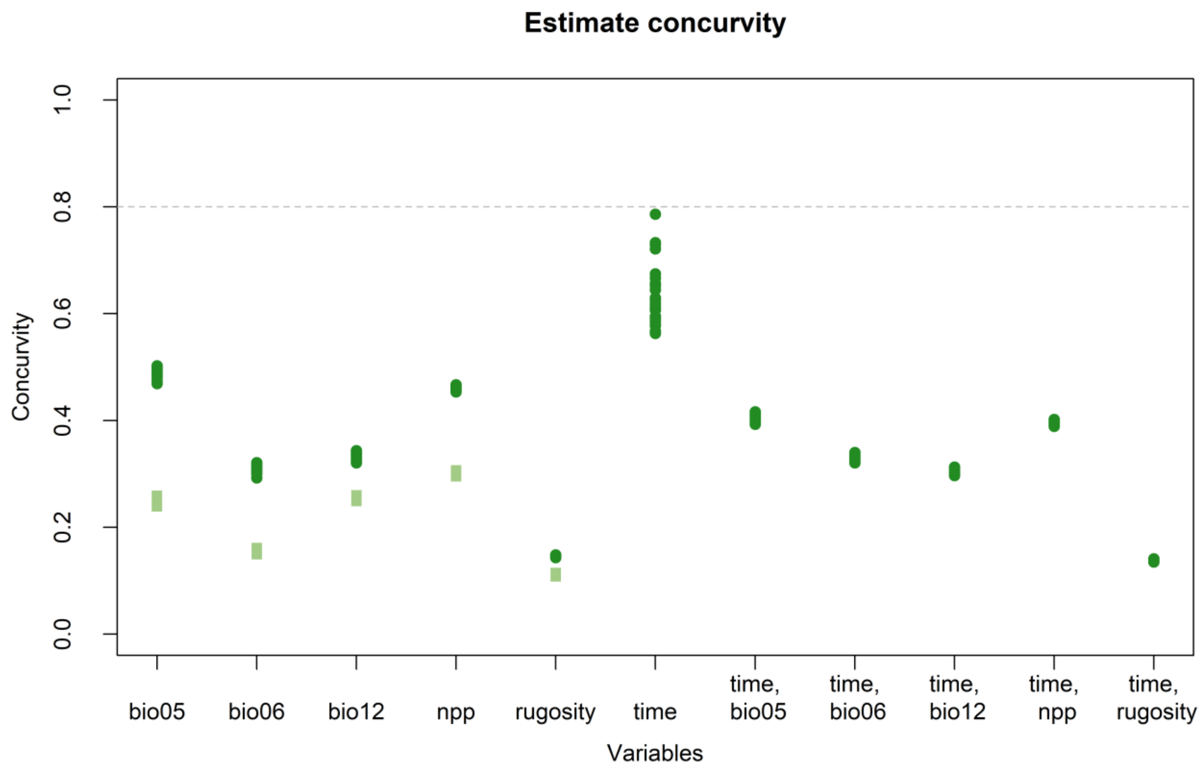

Supplementary figure 5: Measures of estimated concurvity calculated with for the different runs of the constant niche (light points) and full (dark points) models of the horse.

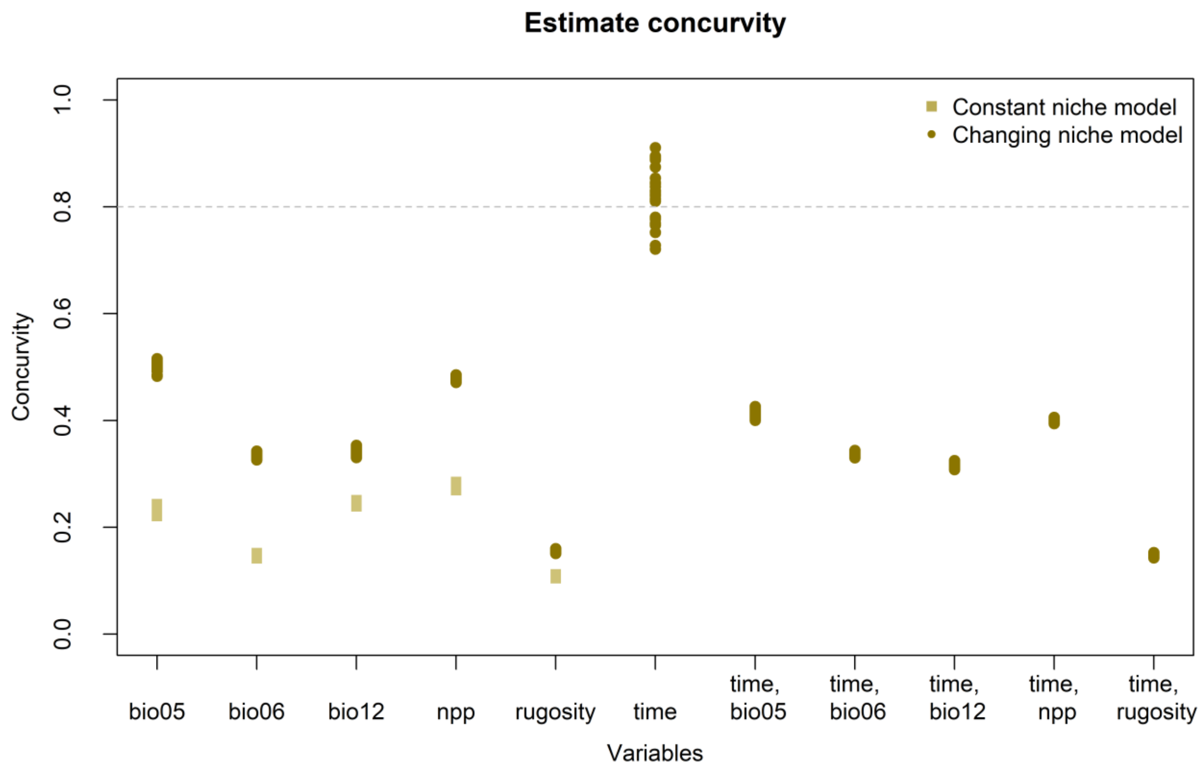

Supplementary figure 6: Measures of estimated concurvity calculated with for the different runs of the constant niche (light points) and full (dark points) models of the aurochs.

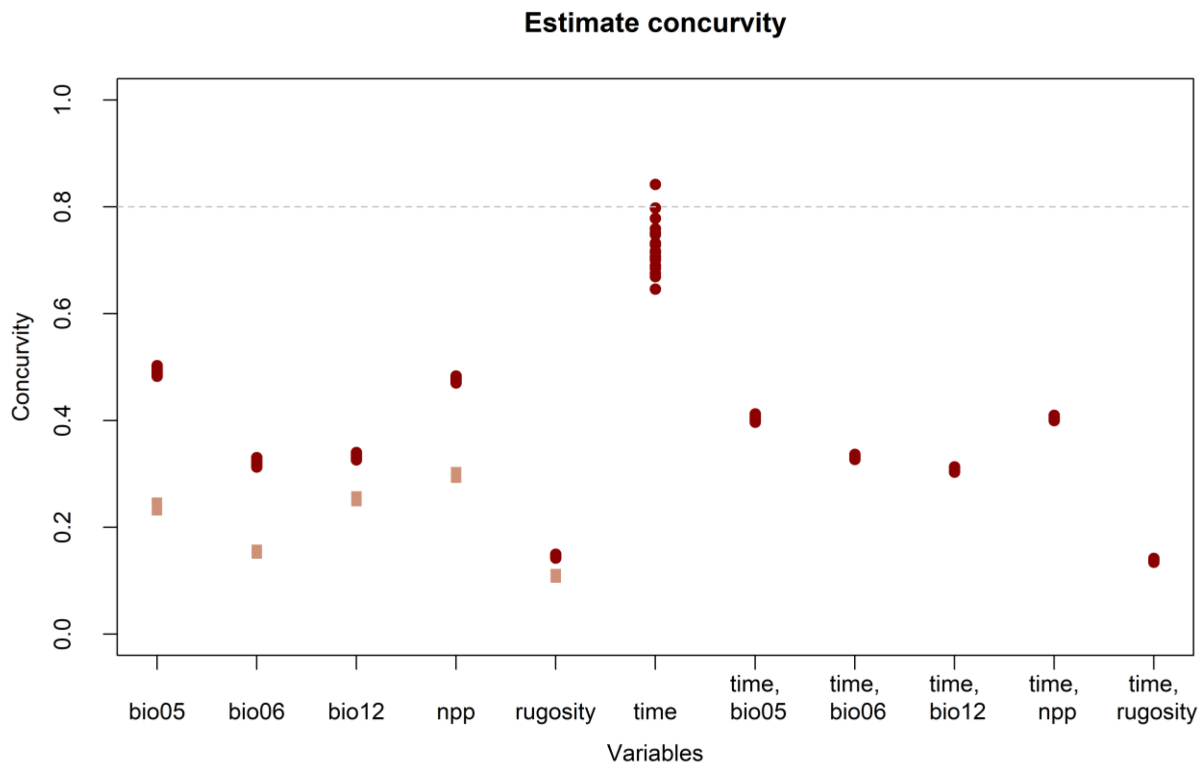

Supplementary figure 7: Measures of estimated concurvity calculated with for the different runs of the constant niche (light points) and full (dark points) models of the deer.

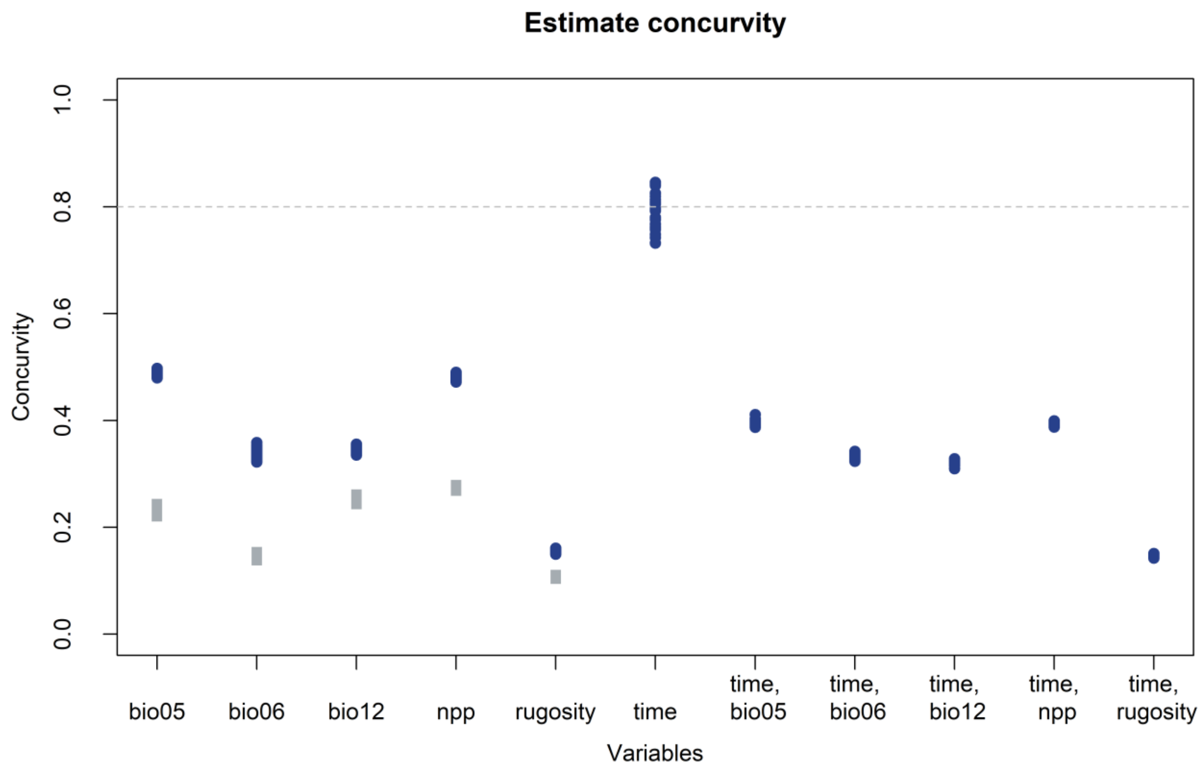

Supplementary figure 8: Measures of estimated concurvity calculated with for the different runs of the constant niche (light points) and full (dark points) models of the wild boar.

Supplementary table 1: Spatial autocorrelation for the horse models, calculated using Moran's I.

	Constant niche model			Changing niche model		
	Moran's I	expected	pval	Moran's I	expected	pval
run 1	0.0032	-0.0001	0	0.0025	-0.0001	0
run 2	0.0041	-0.0001	0	0.0031	-0.0001	0
run 3	0.0041	-0.0001	0	0.0027	-0.0001	0
run 4	0.0042	-0.0001	0	0.0031	-0.0001	0
run 5	0.0035	-0.0001	0	0.0030	-0.0001	0
run 6	0.0040	-0.0001	0	0.0028	-0.0001	0
run 7	0.0033	-0.0001	0	0.0025	-0.0001	0
run 8	0.0044	-0.0001	0	0.0030	-0.0001	0
run 9	0.0042	-0.0001	0	0.0028	-0.0001	0
run 10	0.0038	-0.0001	0	0.0028	-0.0001	0
run 11	0.0042	-0.0001	0	0.0030	-0.0001	0
run 12	0.0034	-0.0001	0	0.0027	-0.0001	0
run 13	0.0032	-0.0001	0	0.0024	-0.0001	0
run 14	0.0043	-0.0001	0	0.0037	-0.0001	0
run 15	0.0039	-0.0001	0	0.0031	-0.0001	0
run 16	0.0033	-0.0001	0	0.0023	-0.0001	0
run 17	0.0039	-0.0001	0	0.0026	-0.0001	0
run 18	0.0041	-0.0001	0	0.0031	-0.0001	0
run 19	0.0041	-0.0001	0	0.0035	-0.0001	0
run 20	0.0034	-0.0001	0	0.0032	-0.0001	0
run 21	0.0038	-0.0001	0	0.0024	-0.0001	0
run 22	0.0037	-0.0001	0	0.0027	-0.0001	0
run 23	0.0041	-0.0001	0	0.0031	-0.0001	0
run 24	0.0035	-0.0001	0	0.0027	-0.0001	0
run 25	0.0035	-0.0001	0	0.0032	-0.0001	0

Supplementary table 2: Spatial autocorrelation for the aurochs models, calculated using Moran's I.

	Constant niche model			Changing niche model		
	Moran's I	expected	pval	Moran's I	expected	pval
run 1	0.0003	-0.0001	9E-02	0.0002	-0.0001	2E-01
run 2	0.0009	-0.0001	0.0003	0.0007	-0.0001	0.004
run 3	0.0004	-0.0001	0.0375	0.0005	-0.0001	3E-02
run 4	0.0011	-0.0001	3E-06	0.0008	-0.0001	7E-04
run 5	0.0008	-0.0001	4E-04	0.0005	-0.0001	2E-02
run 6	0.0006	-0.0001	1E-02	0.0004	-0.0001	6E-02
run 7	0.0004	-0.0001	0.0576	0.0001	-0.0001	4E-01
run 8	0.0009	-0.0001	0.0003	0.0007	-0.0001	4E-03
run 9	0.0002	-0.0001	0.1801	0.0003	-0.0001	0.137
run 10	0.0011	-0.0001	9E-06	0.0009	-0.0001	8E-05
run 11	0.0005	-0.0001	0.0229	0.0001	-0.0001	0.319
run 12	0.0004	-0.0001	0.0674	0.0002	-0.0001	0.201
run 13	0.0010	-0.0001	7E-05	0.0011	-0.0001	1E-05
run 14	0.0004	-0.0001	5E-02	0.0005	-0.0001	3E-02
run 15	0.0004	-0.0001	3E-02	0.0004	-0.0001	0.045
run 16	0.0006	-0.0001	8E-03	0.0004	-0.0001	6E-02
run 17	0.0009	-0.0001	8E-05	0.0009	-0.0001	2E-04
run 18	0.0010	-0.0001	2E-05	0.0005	-0.0001	0.015
run 19	0.0005	-0.0001	2E-02	0.0001	-0.0001	5E-01
run 20	0.0010	-0.0001	3E-05	0.0007	-0.0001	0.001
run 21	0.0009	-0.0001	2E-04	0.0005	-0.0001	0.022
run 22	0.0003	-0.0001	1E-01	0.0001	-0.0001	5E-01
run 23	0.0004	-0.0001	0.0357	0.0002	-0.0001	0.208
run 24	0.0010	-0.0001	6E-05	0.0009	-0.0001	3E-04
run 25	0.0006	-0.0001	4E-03	0.0005	-0.0001	3E-02

Supplementary table 3: Spatial autocorrelation for the deer models, calculated using Moran's I.

	Constant niche model			Changing niche model		
	Moran's I	expected	pval	Moran's I	expected	pval
run 1	0.0034	-0.0001	0	0.0027	-0.0001	0
run 2	0.0034	-0.0001	0	0.0026	-0.0001	0
run 3	0.0033	-0.0001	0	0.0024	-0.0001	0
run 4	0.0034	-0.0001	0	0.0028	-0.0001	0
run 5	0.0031	-0.0001	0	0.0029	-0.0001	0
run 6	0.0038	-0.0001	0	0.0025	-0.0001	0
run 7	0.0032	-0.0001	0	0.0026	-0.0001	0
run 8	0.0036	-0.0001	0	0.0028	-0.0001	0
run 9	0.0034	-0.0001	0	0.0028	-0.0001	0
run 10	0.0041	-0.0001	0	0.0025	-0.0001	0
run 11	0.0031	-0.0001	0	0.0024	-0.0001	0
run 12	0.0032	-0.0001	0	0.0027	-0.0001	0
run 13	0.0030	-0.0001	0	0.0024	-0.0001	0
run 14	0.0032	-0.0001	0	0.0024	-0.0001	0
run 15	0.0035	-0.0001	0	0.0025	-0.0001	0
run 16	0.0035	-0.0001	0	0.0029	-0.0001	0
run 17	0.0031	-0.0001	0	0.0026	-0.0001	0
run 18	0.0032	-0.0001	0	0.0027	-0.0001	0
run 19	0.0036	-0.0001	0	0.0026	-0.0001	0
run 20	0.0029	-0.0001	0	0.0028	-0.0001	0
run 21	0.0031	-0.0001	0	0.0027	-0.0001	0
run 22	0.0029	-0.0001	0	0.0024	-0.0001	0
run 23	0.0033	-0.0001	0	0.0026	-0.0001	0
run 24	0.0034	-0.0001	0	0.0029	-0.0001	0
run 25	0.0038	-0.0001	0	0.0031	-0.0001	0

Supplementary table 4: Spatial autocorrelation for the wild boar models, calculated using Moran's I.

	Constant niche model			Changing niche model		
	Moran's I	expected	pval	Moran's I	expected	pval
run 1	0.0008	-0.0001	0.0002996	0.0003	-0.0001	0.072427
run 2	0.0008	-0.0001	0.0003367	0.0006	-0.0001	0.008438
run 3	0.0010	-0.0001	1.567E-05	0.0010	-0.0001	1.16E-05
run 4	0.0011	-0.0001	2.3E-06	0.0009	-0.0001	0.000142
run 5	0.0011	-0.0001	7.8E-06	0.0010	-0.0001	1.53E-05
run 6	0.0004	-0.0001	0.0669325	0.0004	-0.0001	0.05716
run 7	0.0011	-0.0001	2.1E-06	0.0007	-0.0001	0.001107
run 8	0.0008	-0.0001	0.0003118	0.0003	-0.0001	0.093932
run 9	0.0006	-0.0001	0.0049133	0.0004	-0.0001	0.044622
run 10	0.0010	-0.0001	1.458E-05	0.0003	-0.0001	0.078792
run 11	0.0015	-0.0001	6.942E-10	0.0013	-0.0001	6.94E-08
run 12	0.0009	-0.0001	0.0001843	0.0004	-0.0001	0.035345
run 13	0.0008	-0.0001	0.0006542	0.0007	-0.0001	0.002689
run 14	0.0006	-0.0001	3E-03	0.0004	-0.0001	0.037668
run 15	0.0009	-0.0001	9.802E-05	0.0006	-0.0001	0.006385
run 16	0.0008	-0.0001	0.0004054	0.0006	-0.0001	0.004882
run 17	0.0010	-0.0001	3.783E-05	0.0009	-0.0001	7.13E-05
run 18	0.0011	-0.0001	5.583E-06	0.0010	-0.0001	4.29E-05
run 19	0.0006	-0.0001	0.0055544	0.0009	-0.0001	0.000164
run 20	0.0006	-0.0001	6E-03	0.0003	-0.0001	0.087005
run 21	0.0009	-0.0001	5.853E-05	0.0005	-0.0001	0.012642
run 22	0.0008	-0.0001	0.0009644	0.0003	-0.0001	0.09211
run 23	0.0007	-0.0001	0.0020047	0.0007	-0.0001	0.001196
run 24	0.0007	-0.0001	0.0015343	0.0005	-0.0001	0.014709
run 25	0.0011	-0.0001	3.233E-06	0.0007	-0.0001	0.001226

Supplementary table 5: Three statistics to evaluate the horse models: AIC (Akaike Information Criterion), Nagelkerke R2, deviance explained. All favour the changing niche model.

		AIC		Nagelkerke R2		Deviance explained	
	Delta AIC constant-changing niche	Costant niche	Changing niche	Costant niche	Changing niche	Costant niche	Changing niche
run 1	265	5332	5068	0.295	0.355	0.276	0.334
run 2	279	5342	5063	0.294	0.357	0.274	0.335
run 3	270	5363	5093	0.29	0.352	0.271	0.33
run 4	247	5343	5096	0.293	0.35	0.274	0.328
run 5	269	5391	5122	0.287	0.347	0.267	0.326
run 6	270	5387	5117	0.287	0.348	0.268	0.327
run 7	264	5368	5104	0.29	0.349	0.271	0.327
run 8	283	5341	5058	0.294	0.356	0.274	0.335
run 9	265	5346	5081	0.293	0.352	0.274	0.331
run 10	284	5357	5073	0.291	0.355	0.272	0.333
run 11	276	5355	5080	0.292	0.354	0.272	0.333
run 12	276	5327	5051	0.296	0.358	0.277	0.337
run 13	283	5355	5071	0.292	0.354	0.272	0.332
run 14	273	5373	5100	0.289	0.35	0.27	0.329
run 15	264	5341	5077	0.294	0.354	0.274	0.332
run 16	256	5363	5108	0.29	0.349	0.271	0.328
run 17	264	5371	5107	0.29	0.349	0.27	0.328
run 18	254	5368	5114	0.29	0.347	0.27	0.326
run 19	284	5358	5073	0.291	0.355	0.272	0.333
run 20	267	5346	5079	0.293	0.354	0.274	0.333
run 21	272	5349	5077	0.293	0.354	0.273	0.332
run 22	275	5355	5080	0.292	0.354	0.272	0.333
run 23	271	5376	5104	0.289	0.349	0.269	0.327
run 24	256	5321	5066	0.297	0.354	0.277	0.333
run 25	285	5347	5062	0.293	0.356	0.273	0.335

Supplementary table 6: Three statistics to evaluate the aurochs models: AIC (Akaike Information Criterion), Nagelkerke R2, deviance explained. All favour the changing niche model.

		AIC		Nagelkerke R2		Deviance explained	
	Delta AIC constant-changing niche	Costant niche	Changing niche	Costant niche	Changing niche	Costant niche	Changing niche
run 1	187	3058	2871	0.292	0.369	0.273	0.347
run 2	206	3096	2890	0.282	0.365	0.263	0.343
run 3	181	3057	2876	0.292	0.362	0.273	0.34
run 4	203	3081	2878	0.284	0.367	0.265	0.345
run 5	179	3078	2899	0.287	0.362	0.268	0.34
run 6	199	3074	2876	0.288	0.369	0.269	0.347
run 7	178	3079	2901	0.287	0.36	0.268	0.339
run 8	189	3086	2897	0.285	0.363	0.266	0.341
run 9	173	3065	2893	0.29	0.359	0.271	0.338
run 10	177	3054	2877	0.293	0.361	0.274	0.34
run 11	176	3083	2906	0.286	0.357	0.266	0.335
run 12	194	3091	2897	0.284	0.363	0.265	0.341
run 13	176	3073	2897	0.288	0.358	0.269	0.336
run 14	183	3058	2875	0.292	0.362	0.273	0.34
run 15	195	3062	2867	0.291	0.369	0.272	0.347
run 16	171	3089	2918	0.284	0.356	0.265	0.334
run 17	181	3060	2879	0.292	0.361	0.272	0.339
run 18	176	3066	2890	0.29	0.359	0.271	0.338
run 19	184	3070	2886	0.289	0.363	0.27	0.341
run 20	191	3072	2881	0.288	0.37	0.269	0.348
run 21	185	3064	2879	0.291	0.363	0.271	0.341
run 22	188	3075	2887	0.288	0.362	0.269	0.34
run 23	184	3083	2899	0.286	0.362	0.267	0.34
run 24	188	3079	2891	0.287	0.365	0.268	0.343
run 25	203	3061	2858	0.291	0.372	0.272	0.35

Supplementary table 7: Three statistics to evaluate the deer models: AIC (Akaike Information Criterion), Nagelkerke R2, deviance explained. All favour the changing niche model.

	Delta AIC constant-changing niche	Costant niche	Changing niche	Costant niche	Changing niche	Costant niche	Changing niche
run 1	262	5963	5701	0.324	0.372	0.303	0.35
run 2	266	5986	5720	0.321	0.37	0.3	0.348
run 3	256	5990	5734	0.321	0.368	0.3	0.346
run 4	268	5968	5701	0.323	0.374	0.303	0.352
run 5	262	5964	5703	0.324	0.372	0.303	0.35
run 6	270	5998	5728	0.32	0.37	0.299	0.348
run 7	262	5993	5731	0.32	0.368	0.3	0.346
run 8	260	5982	5722	0.321	0.37	0.301	0.348
run 9	257	5972	5715	0.323	0.371	0.302	0.349
run 10	278	5961	5683	0.324	0.375	0.303	0.352
run 11	264	5956	5692	0.325	0.374	0.304	0.352
run 12	278	5965	5687	0.324	0.375	0.303	0.353
run 13	267	5989	5723	0.321	0.371	0.3	0.349
run 14	273	5963	5690	0.324	0.374	0.303	0.352
run 15	282	5969	5686	0.323	0.375	0.302	0.353
run 16	294	5990	5696	0.32	0.374	0.3	0.352
run 17	265	5983	5718	0.321	0.37	0.301	0.348
run 18	255	5997	5742	0.32	0.367	0.299	0.345
run 19	265	6004	5739	0.319	0.369	0.298	0.347
run 20	280	5973	5693	0.323	0.375	0.302	0.353
run 21	254	5972	5718	0.323	0.371	0.302	0.349
run 22	271	5989	5718	0.321	0.372	0.3	0.349
run 23	271	5974	5703	0.323	0.375	0.302	0.352
run 24	269	5966	5697	0.323	0.373	0.303	0.351
run 25	278	5970	5692	0.323	0.374	0.302	0.352

Supplementary table 8: Three statistics to evaluate the wild boar models: AIC (Akaike Information Criterion), Nagelkerke R2, deviance explained. All favour the changing niche model.

	Delta AIC constant-changing niche	Costant niche	Changing niche	Costant niche	Changing niche	Costant niche	Changing niche
run 1	149	3069	2920	0.338	0.395	0.317	0.373
run 2	160	3074	2914	0.337	0.401	0.316	0.378
run 3	153	3080	2927	0.335	0.397	0.314	0.374
run 4	155	3066	2912	0.339	0.4	0.318	0.377
run 5	153	3053	2901	0.341	0.405	0.32	0.382
run 6	119	3062	2943	0.34	0.388	0.319	0.366
run 7	154	3043	2890	0.344	0.407	0.323	0.384
run 8	135	3090	2955	0.333	0.391	0.312	0.368
run 9	114	3055	2942	0.341	0.386	0.32	0.363
run 10	155	3060	2905	0.34	0.407	0.319	0.384
run 11	127	3084	2957	0.335	0.385	0.314	0.363
run 12	117	3043	2926	0.344	0.397	0.323	0.374
run 13	142	3062	2920	0.34	0.402	0.318	0.379
run 14	150	3068	2919	0.338	0.4	0.317	0.378
run 15	132	3056	2925	0.341	0.391	0.319	0.368
run 16	164	3060	2895	0.34	0.409	0.319	0.386
run 17	135	3064	2929	0.339	0.393	0.318	0.37
run 18	126	3068	2942	0.338	0.386	0.317	0.364
run 19	158	3038	2880	0.345	0.409	0.324	0.386
run 20	148	3086	2937	0.334	0.395	0.313	0.372
run 21	121	3058	2937	0.34	0.386	0.319	0.363
run 22	153	3072	2918	0.337	0.4	0.316	0.378
run 23	160	3078	2918	0.336	0.403	0.315	0.38
run 24	140	3043	2903	0.344	0.399	0.323	0.377
run 25	152	3067	2915	0.338	0.402	0.317	0.379

Supplementary table 9: Boyce continuous index (BCI) for each repetition (=run) of the changing niche models. There are no values below the acceptance threshold of 0.8.

	Horse	Aurochs	Deer	Wildboar
run 1	0.98	0.89	0.98	0.98
run 2	0.97	0.98	1.00	0.97
run 3	0.98	0.91	0.99	0.99
run 4	0.98	0.96	0.96	0.97
run 5	0.97	0.95	0.97	0.99
run 6	0.94	0.92	0.93	0.93
run 7	0.99	0.98	0.94	0.99
run 8	0.99	0.80	0.96	0.94
run 9	0.98	0.98	0.96	0.90
run 10	0.99	0.94	0.99	0.98
run 11	0.98	0.98	0.98	0.90
run 12	0.98	0.93	0.98	0.97
run 13	0.97	0.96	0.97	0.95
run 14	0.98	0.97	0.97	0.97
run 15	0.98	0.98	0.98	0.94
run 16	0.99	0.98	0.97	0.96
run 17	0.98	0.98	0.97	0.98
run 18	0.99	0.97	0.98	0.95
run 19	0.98	0.95	0.98	0.95
run 20	0.93	0.93	0.96	0.97
run 21	0.99	0.91	0.98	0.98
run 22	0.96	0.89	0.97	0.94
run 23	0.92	0.95	0.96	0.99
run 24	0.98	0.94	0.99	0.98
run 25	0.98	0.95	0.98	0.98
