# Supplementary material for: Following the niche: the differential impact of the last glacial maximum on four European ungulates
Source: Commun Biol. 2022 Sep 29;5:1038. doi: 10.1038/s42003-022-03993-7 (PMC9523052; doi:10.1038/s42003-022-03993-7)
Supplement: Supplementary file 3 — Reporting Summary [file 42003_2022_3993_MOESM3_ESM.pdf]

nature research

Corresponding author(s): Michela Leonardi

Last updated by author(s): 19/08/2022

Reporting Summary

Nature Research wishes to improve the reproducibility of the work that we publish. This form provides structure for consistency and transparency in reporting. For further information on Nature Research policies, see our Editorial Policies and the Editorial Policy Checklist.

Statistics

For all statistical analyses, confirm that the following items are present in the figure legend, table legend, main text, or Methods section.

n/a	Confirmed	
☐	☒	The exact sample size (n) for each experimental group/condition, given as a discrete number and unit of measurement
☐	☒	A statement on whether measurements were taken from distinct samples or whether the same sample was measured repeatedly
☐	☒	The statistical test(s) used AND whether they are one- or two-sided<br>*Only common tests should be described solely by name; describe more complex techniques in the Methods section.*
☐	☒	A description of all covariates tested
☐	☒	A description of any assumptions or corrections, such as tests of normality and adjustment for multiple comparisons
☐	☒	A full description of the statistical parameters including central tendency (e.g. means) or other basic estimates (e.g. regression coefficient) AND variation (e.g. standard deviation) or associated estimates of uncertainty (e.g. confidence intervals)
☐	☒	For null hypothesis testing, the test statistic (e.g. F, t, r) with confidence intervals, effect sizes, degrees of freedom and P value noted<br>*Give P values as exact values whenever suitable.*
☐	☒	For Bayesian analysis, information on the choice of priors and Markov chain Monte Carlo settings
☐	☒	For hierarchical and complex designs, identification of the appropriate level for tests and full reporting of outcomes
☐	☒	Estimates of effect sizes (e.g. Cohen's d, Pearson's r), indicating how they were calculated

Our web collection on statistics for biologists contains articles on many of the points above.

Software and code

Policy information about availability of computer code

Data collection	no software was used for data collection
Data analysis	the whole code used for data analysis is available in the supplementary materials at the link https://figshare.com/s/383a2902a29ca1860550 in the form of R markdown file. It has been run in R 4.2.1

For manuscripts utilizing custom algorithms or software that are central to the research but not yet described in published literature, software must be made available to editors and reviewers. We strongly encourage code deposition in a community repository (e.g. GitHub). See the Nature Research guidelines for submitting code & software for further information.

Data

Policy information about availability of data

All manuscripts must include a data availability statement. This statement should provide the following information, where applicable:

- Accession codes, unique identifiers, or web links for publicly available datasets
- A list of figures that have associated raw data
- A description of any restrictions on data availability

All data analysed during this study can be accessed at the link https://figshare.com/s/383a2902a29ca1860550

Field-specific reporting

Please select the one below that is the best fit for your research. If you are not sure, read the appropriate sections before making your selection.

☐ Life sciences ☐ Behavioural & social sciences ☒ Ecological, evolutionary & environmental sciences

For a reference copy of the document with all sections, see nature.com/documents/nr-reporting-summary-flat.pdf

Ecological, evolutionary & environmental sciences study design

All studies must disclose on these points even when the disclosure is negative.

Study description	SDM using GAMs of time series of radiocarbon dates
Research sample	We collected from the literature and available databases a dataset of radiocarbon dates from Western Eurasia (West of 60°E and North of 37°N) either obtained from remains of Equus ferus, Bos primigenius, Cervus elaphus, or Sus scrofa, or from archaeological layers where the species has been observed.
Sampling strategy	Comprehensive survey of all available radiocarbon dates available in public databases (ORAU, STAGE3, Radiocarbon Paleaeolithic European Database, EUROEVOL, Radon, Radonb, Archaeology Data Service, KIKIRPA, Banadora, CARD, CONTEXT, CalPal database) and publications.
Data collection	Michela Leonardi collected the dataset of radiocarbon dates associated to remains of the species, Francesco Boschin recorded from the literature presences of the species in archeological assemblages, to which Michela Leonardi coupled radiocarbon dates associated to the same archaeological layer (when available).
Timing and spatial scale	Western Eurasia (West of 60°E and North of 37°N) , 47 to 7.5 thousand of years ago
Data exclusions	We excluded any record fitting one or more of the following conditions: unreliable; not in accord with the expected chronology of their archaeological layer; without a reported standard error; available only as terminum ante/post quem.<br>All dates were calibrated with OxCal 5 version 4.4 using the IntCal20 curve 48, and we further excluded any record for which calibration resulted in an error.
Reproducibility	The whole data and code used for the analyses is made available in the supplementary materials at the link https://figshare.com/s/383a2902a29ca1860550
Randomization	Models were validated by generating 10 set of pseudoabsences.
Blinding	Not applicable as there was no observational component

Did the study involve field work? ☐ Yes ☒ No

Reporting for specific materials, systems and methods

We require information from authors about some types of materials, experimental systems and methods used in many studies. Here, indicate whether each material, system or method listed is relevant to your study. If you are not sure if a list item applies to your research, read the appropriate section before selecting a response.

Materials & experimental systems

n/a	Involved in the study	
☒	☐	Antibodies
☒	☐	Eukaryotic cell lines
☒	☐	Palaeontology and archaeology
☒	☐	Animals and other organisms
☒	☐	Human research participants
☒	☐	Clinical data
☒	☐	Dual use research of concern

Methods

n/a	Involved in the study	
☒	☐	ChIP-seq
☒	☐	Flow cytometry
☒	☐	MRI-based neuroimaging
